# Revitalizing the Dull: A Classic Approach to Nonvital Tooth Bleaching

**DOI:** 10.1155/crid/5318875

**Published:** 2025-11-03

**Authors:** Priyanka Bhojwani, Anuja Ikhar, Aditya Patel, Manoj Chandak, Shweta Sedani

**Affiliations:** Department of Conservative Dentistry and Endodontics, Datta Meghe Institute of Higher Education and Research, Wardha, Maharashtra, India

## Abstract

Nonvital tooth bleaching is a treatment designed to enhance the cosmetic appearance of discolored teeth that have lost vitality. This procedure has become increasingly popular in contemporary dentistry due to its effectiveness. Among the various chemical agents used for this purpose, sodium perborate, when combined with hydrogen peroxide, has proven to be one of the most efficient solutions for achieving noticeable whitening outcomes. Sodium perborate is a stable oxidizing agent, frequently mixed with hydrogen peroxide to form a bleaching paste. This paste is then placed directly into the pulp chamber of a nonvital tooth. The oxidation reaction that occurs helps to break down organic pigments within the tooth, thereby reducing discoloration without causing harm to the surrounding dental tissues. Research has shown that the combined use of sodium perborate and hydrogen peroxide can effectively restore a tooth's natural color, with a low incidence of complications such as root resorption or external cervical resorption. This method has been proven to be both clinically successful and generally safe, though it is crucial to carefully monitor the concentration of the bleaching agents and the duration of their application to prevent excessive bleaching or harm to the tooth's structure. Nonvital tooth bleaching has emerged as a valuable technique for teeth that have undergone root canal treatment, providing a noninvasive and efficient alternative to more extensive restorative procedures. This review discusses the underlying mechanisms, efficacy, safety, and potential risks associated with nonvital tooth whitening using sodium perborate and hydrogen peroxide, offering a thorough overview of its role in modern dental practice.

## 1. Introduction

As teeth erupt, the anatomy of the apical foramen undergoes changes because root formation is not yet complete. Root development and closure of the apex typically continue for up to 3 years after eruption. Patients with incomplete apical development present a unique clinical challenge due to large, open apices and thin, divergent dentinal walls, which increase the risk of fracture. Traditionally, clinicians used prolonged calcium hydroxide therapy over several months to stimulate apical barrier formation. More recently, immediate placement of a mineral trioxide aggregate (MTA) plug has been adopted to create an apical barrier. Inadequate root development that prevents formation of a conical canal taper is commonly described as a “blunderbuss” canal [1].

There are different types of open apices. Nonblunderbuss apices can be broad and cylindrical, maintaining a uniform width, or tapered and convergent, where the apex narrows gradually toward the tip. In contrast, a blunderbuss apex is funnel-shaped, typically wider at the tip than the coronal portion of the canal.

Open apices may arise due to several causes, including caries involving the pulp, extensive resorption of a mature apex following orthodontic treatment, periapical pathosis, or trauma resulting in pulp necrosis [2].

The presence of an open apex presents two primary challenges. First, it disrupts the normal crown-to-root ratio, which can lead to tooth mobility. Second, it complicates achieving an effective apical seal with conventional root canal filling techniques [3].

Nonvital tooth bleaching is a procedure used to whiten discolored teeth that have previously undergone root canal therapy. Teeth lose vitality due to the accumulation of bacteria, blood, and other materials within the pulp chamber, causing discoloration over time. Nonvital bleaching provides a reliable and safe method to restore the tooth's original color [4].

Sodium perborate is commonly used in nonvital tooth bleaching. This chemical decomposes to release oxygen when applied inside the tooth, breaking down internal stains and reducing discoloration without damaging the surrounding enamel. It is often mixed with hydrogen peroxide or water to form a bleaching paste that is placed in the pulp chamber and temporarily sealed. Over a few days, this bleaching agent whitens the tooth, producing a more uniform and esthetic result [5].

This technique is especially beneficial for patients who have undergone root canal therapy and are concerned about the darkening of the treated tooth [6]. When performed by a skilled dentist, nonvital bleaching with sodium perborate is minimally invasive and generally well tolerated and can produce long-lasting results with minimal risk (Table 1) [7, 8].

## 2. Case Presentation

### 2.1. Patient Information

**Figure 1:**
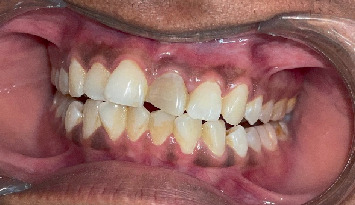
Preoperative intraoral photograph.

**Figure 2:**
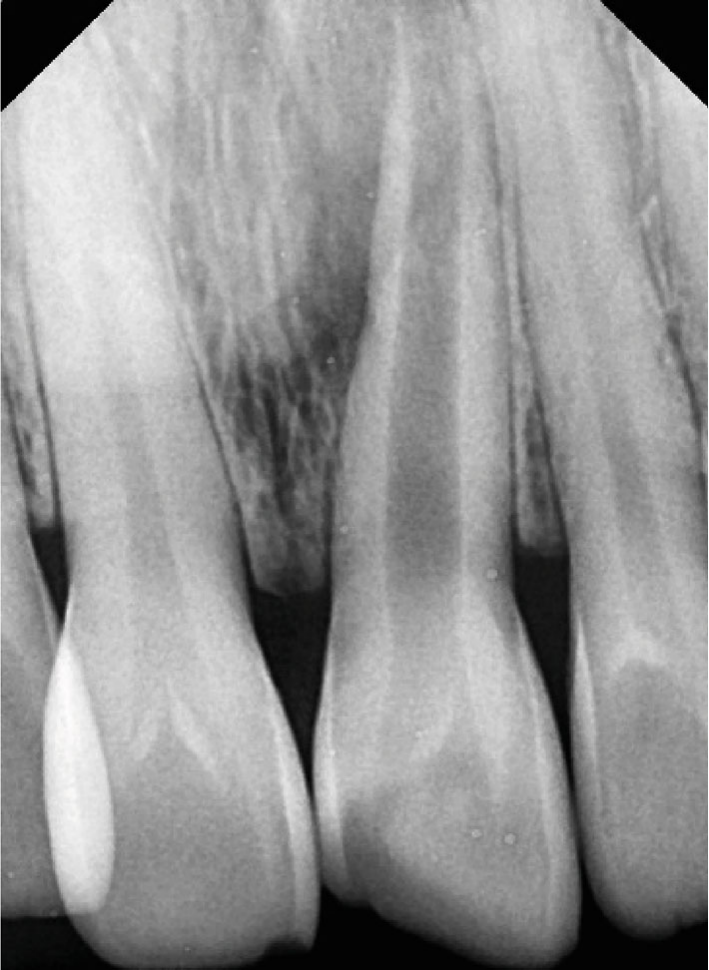
Preoperative radiograph.

The Sharad Pawar Dental College and Hospital outpatient department referred a 39-year-old female patient presenting with a discolored and fractured left upper anterior tooth, as shown in Figures 1 and 2.

### 2.2. Timeline

The timeline is since 15 days.

### 2.3. Diagnosis Assessment

Based on clinical and radiographic evaluation, a preliminary diagnosis of pulpal necrosis with an Ellis Class II fracture of the left upper anterior tooth was made. The patient reported a history of trauma 20 years ago. Clinical examination revealed crowded teeth. Orthodontic treatment was offered to address the crowding; however, the patient declined. A final diagnosis of pulpal necrosis with an Ellis Class II fracture in Tooth #21 was established.

### 2.4. Informed Consent

Informed consent was taken prior to beginning the treatment.

### 2.5. Therapeutic Interventions

The patient was administered 2% xylocaine with 1:80,000 adrenaline (Neon Laboratories Limited, Mumbai, Maharashtra, India). Given the patient's medical history, the procedure utilized high-power suction. A round BR-45 bur (MANI Inc., Vietnam) was used to create the access cavity, and then, a safe end bur EX-24 (MANI Inc., Vietnam) was applied to further extend the cavity via lateral movements. After the pulp tissue from the chamber was completely removed, a single opening was noted.

**Figure 3:**
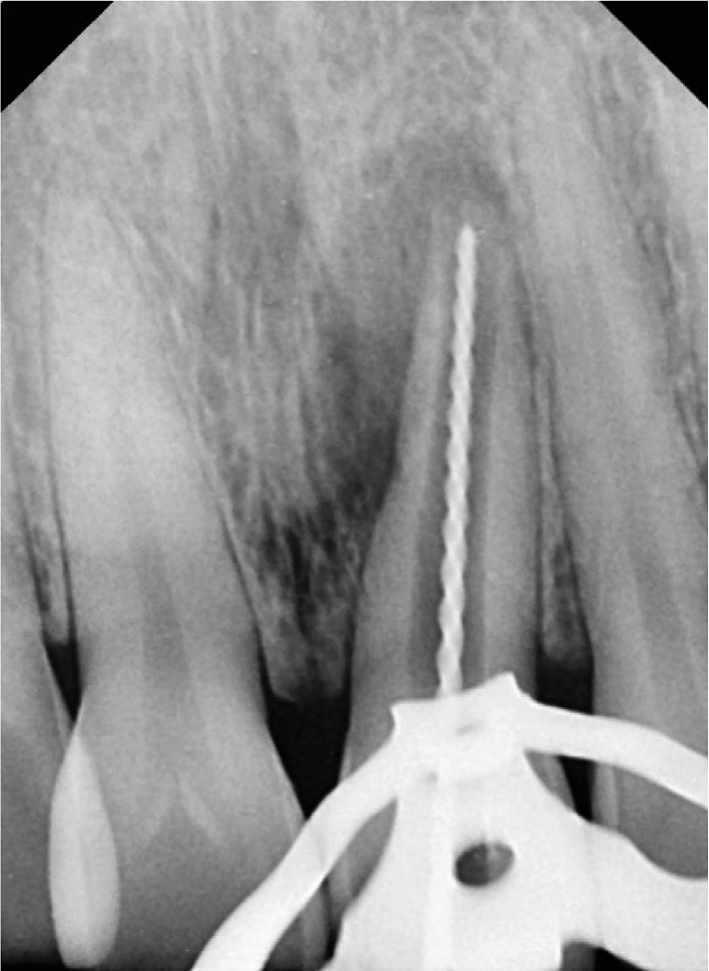
Working length determination.

A No. 10 K-file (MANI Inc., Vietnam) was employed to identify the openings of the root canal, and the working length was assessed via an electronic apex locator (J. Morita Corporation, Japan). It was re-evaluated on the radiograph as shown in Figure 3.

**Figure 4:**
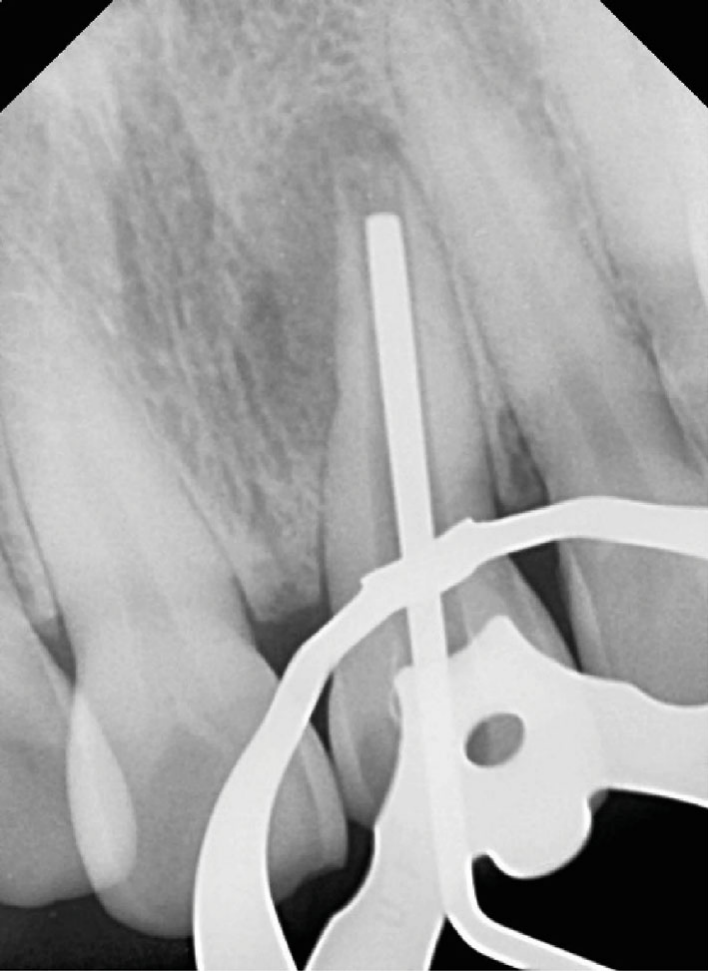
Plugger size selection.

**Figure 5:**
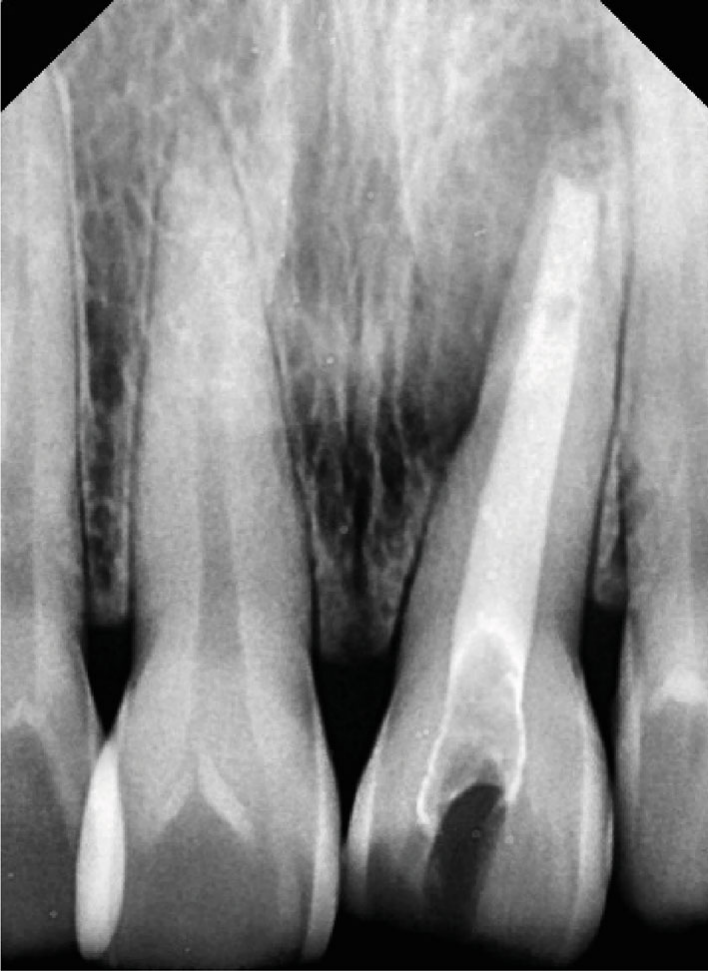
MTA obturation.

Biomechanical preparation of the root canal was carried out using hand files up to size 55 K with a 2% taper (MANI Inc., Vietnam). The canals were irrigated alternately with 3% NaOCl (Vishal Dentocare, India) and 0.9% saline (Parenteral Drugs (India) Ltd.). A temporary restorative material (Cavit G 3M ESPE, Europe Germany) was applied, and the patient was instructed to return for a follow-up after 4 days. At the second appointment, the temporary material was removed, and the canal was sonically activated with 17% neo ethylenediaminetetraacetic acid (Prevest DenPro India). The canals were irrigated again with 3% NaOCl and 0.9% saline. Plugger (plugger woodpecker, China) fit was checked radiographically as shown in Figure 4. The canal was obturated using MTA (SafeEndo BioStructure Vadodara, Gujarat, India) as shown in Figure 5.

Moist cotton pellet was placed, temporary restoration was placed, and the patient was recalled after 3 days.

**Figure 6:**
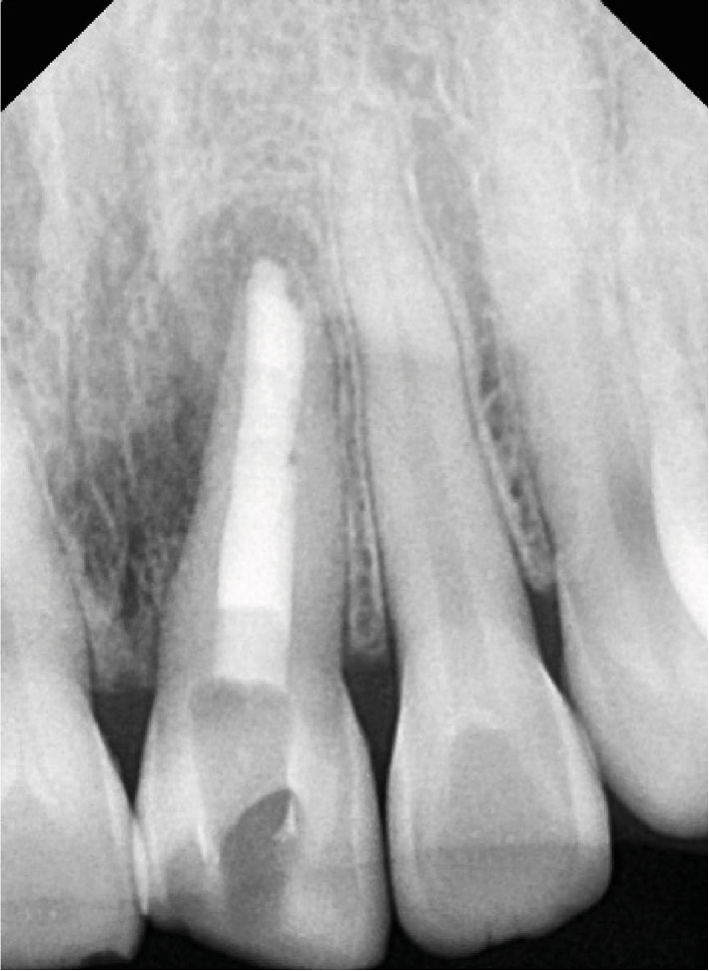
GIC base applied for intracoronal bleaching.

On the fourth visit, temporary restoration was removed, cotton was removed, and a layer of glass ionomer cement (Gc Corporation, Tokyo, Japan) as shown in Figure 6 was applied over the MTA.

**Figure 7:**
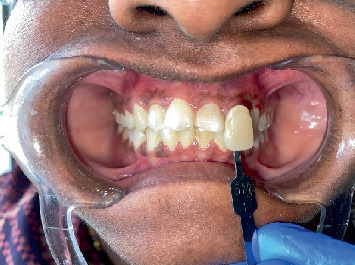
Shade selection done before bleaching.

**Figure 8:**
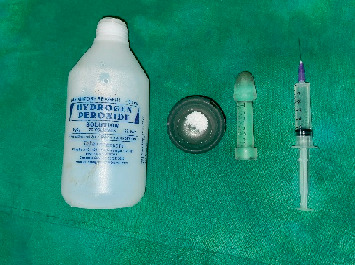
Sodium perborate and hydrogen peroxide were used as a bleaching agent.

**Figure 9:**
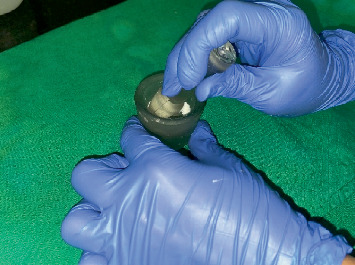
Mixed to make a thick paste.

Shade selection done before bleaching using Vita Classical Shade Guide (VITA Zahnfabrik, Germany) was as shown in Figure 7. Thirty percent hydrogen peroxide (tekay products, Mumbai) (as shown in Figure 8) was mixed with sodium perborate (HiMedia, United States) and was mixed in a mortar and pestle (as shown in Figure 9) and was placed inside the pulp chamber and compressed with a damp cotton pellet. The access cavity was sealed with Cavit. Due to minimal color improvement, the procedure was repeated after 1 week. Following the second application, significant tooth whitening was achieved. To complete the treatment, the access cavity was restored using a composite resin and bonding system (GC Corporation, Tokyo, Japan).

### 2.6. Follow-Up and Outcomes

**Figure 10:**
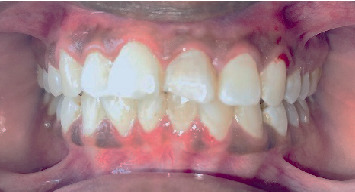
Postoperative clinical image.

The postoperative clinical image is shown in Figure 10. Telephonic follow-up was maintained; the patient was satisfied with the treatment outcome.

## 3. Discussion

The pulp experiences necrosis in response to trauma or cavities, which stops dentin formation and prevents additional root growth. The outcome is an abnormally big apical aperture, which indicates that the root is still young. Previously referred to as a blunderbuss canal, this condition is called an open apex [9, 10].

According to Gupta et al. [11], two patients stopped receiving therapy with the walking bleach technique, mostly because they were unhappy with the need for several visits. According to the same study, longer treatment times were needed for patients who were older or had more persistent discoloration. The number of appointments required was unaffected by the degree of discoloration, though.

Conversely, there are several disadvantages to the combination bleaching method. In particular, it necessitates greater patient cooperation and keeps the hole open during the procedure, increasing the risk of fracture. This entails going back to the office for the final restoration and following the treatment protocol throughout the procedure [7, 8].

According to Bizhang et al. [8], the combination bleaching procedure and the walking bleach method produced no discernible difference by the 6-month mark, and both were determined to be equally successful. One month following the end of therapy, Bersezio et al. [6] saw a little color regression. Bizhang et al. [8] also noted this at 6 months, and Lise et al. [7] did the same during a 1-year follow-up. The tooth's rehydration is probably the cause of this regression. To counteract this chromatic regression, some researchers recommend overwhitening [6]. With intracoronary bleaching, discoloration recurrence is somewhat prevalent and is impacted by the amount of time that has passed since the end of treatment. After 2, 5, and 8 years, the walking bleach approach has been found to have failure rates ranging from 10% to 49%.

Tooth shade determination in this case was performed using the VITA Classical Shade Guide, a widely accepted and commonly used visual shade selection system in clinical dentistry. This guide offers several advantages: it is clinically familiar to most dental professionals, is simple to use, and provides a quick visual reference for selecting shades that approximate natural tooth color. Its broad use also facilitates standardized communication between clinicians and dental laboratories. However, the VITA Classical Shade Guide also has limitations. It is a subjective technique that depends heavily on the clinician's visual perception, which may vary between individuals. Additionally, ambient lighting conditions, the patient's surrounding environment, and even operator fatigue can influence shade selection accuracy. Furthermore, the guide offers a limited range of shades, which may not fully capture the nuances of natural tooth coloration, especially in cases requiring high esthetic demands. Despite these limitations, its practicality and accessibility make it a preferred tool in everyday clinical practice.

Lise et al. [7] found no instances of root resorption at the 1-year follow-up following treatment completion, but other studies did not include this particular assessment. Root resorption is still a possible side effect of internal tooth bleaching and should be minimized; research suggests that cervical root resorption can be prevented or reduced by placing a cervical biomechanical barrier; in the walking bleach, 3 mm of the canal filling material should be removed apical to the amelocemental junction, followed by a layer of glass ionomer cement [12, 13]. This barrier's function is to stop the bleaching agent from spreading into the periapical area through the dentinal tubules [7, 8].

A slightly acidic environment increases the activity of osteoclasts and polymorphonuclear leukocytes. By encouraging acidic hydrolysis, they demineralize hard tissues and prevent the production of new hard tissue. Therefore, if the pH of the cervical periodontal ligament's milieu changes, external cervical root resorption might happen. A 30% hydrogen peroxide solution, for instance, has a pH range of 2–3, whereas 10% carbamide peroxide raises the pH and offers more clinical safety by dissolving into 3.35% hydrogen peroxide and 6.65% urea solution [8]. Some researchers have proposed sodium perborate, which has a pH of 10–12, as a hydrogen peroxide substitute or as a combination agent with hydrogen peroxide at low concentrations. This combination is believed to lessen the chance of external root resorption by balancing the pH shift brought on by hydrogen peroxide [14, 15].

Importantly, the concentrations of bleaching agents used in the walking bleach technique are 20% hydrogen peroxide [7]. This discrepancy is probably caused by how frequently the bleaching agent is changed. The walking bleach technique requires greater concentrations since the agent is changed weekly.

Regulations requiring the evaluation of cosmetic compounds categorized as carcinogenic, mutagenic, or hazardous to reproduction, taking into account all potential exposure sources, were introduced by the European Union in 2009 for safety concerns. As a result of the borate family's classification, the European Union outlawed its use in cosmetic products, including tooth-whitening agents, on December 1, 2010. The European Union also forbade the use of hydrogen peroxide and other substances or mixes that emit hydrogen peroxide, including carbamide peroxide, in concentrations higher than 6% in order to guarantee clinical safety. Furthermore, it was mandated that, starting in 2011, only dentists could sell tooth whitening solutions with hydrogen peroxide concentrations between 0.1% and 6%, whereas over-the-counter treatments must not contain more than 0.1% hydrogen peroxide [2].

### 3.1. Patient Perspective

The patient was highly satisfied with the treatment as the treatment provided an esthetic outcome painlessly.

## 4. Conclusion

In conclusion, nonvital bleaching techniques represent a valuable and effective approach in enhancing the esthetics of discolored teeth with pulp-related issues. As such, it is essential to emphasize the significance of proper diagnosis, treatment planning, and patient communication in the success of nonvital bleaching procedures. Each case is unique, and a thorough assessment by the dental practitioner is crucial to determine the most suitable technique, concentration, and duration.

## Figures and Tables

**Table 1:** Summary of vital and nonvital bleaching.

**Tooth type**	**Whitening technique**	**Method**	**Common agents**	**Indications**
Vital teeth	In-office bleaching	Light/laser-activated bleaching	Hydrogen peroxide (25%–40%)	Rapid whitening under professional supervision
	At-home bleaching (professional)	Custom trays with bleaching gel	Carbamide peroxide (10%–20%)	Mild to moderate discoloration
	Over-the-counter (OTC) products	Whitening strips, toothpaste, rinses	Peroxide-based or abrasives	Mild surface stains
	Microabrasion	Mechanical + chemical removal of stains	Abrasive + acid slurry	Superficial enamel discoloration (e.g., fluorosis)

Nonvital teeth	Internal (intracoronal) bleaching	Placement of bleaching agent inside pulp chamber	Sodium perborate + hydrogen peroxide	Discoloration after root canal treatment
	Walking bleach technique	Sealed bleaching agent left in the tooth	Sodium perborate + water/peroxide	Gradual internal whitening for endodontically treated teeth

## Data Availability

The authors have nothing to report.
